# Supplementary material for: Biomedical knowledge graph learning for drug repurposing by extending guilt-by-association to multiple layers
Source: Nat Commun. 2023 Jun 15;14:3570. doi: 10.1038/s41467-023-39301-y (PMC10272215; doi:10.1038/s41467-023-39301-y)
Supplement: Supplementary file 1 — Supplementary Information [file 41467_2023_39301_MOESM1_ESM.pdf]

SUPPLEMENTARY INFORMATION FOR

Biomedical knowledge graph learning for drug repurposing by extending guilt-by-association to multiple layers

Bang et al., *Nature Communications* 2023

*Corresponding author. Email: sunkim.bioinfo@snu.ac.kr

Supplementary Information includes:

Supplementary Method 1.

Supplementary Figures 1 to 6.

Supplementary Table 1 to 7.

Supplementary References

Table of Contents

Supplementary Methods

Supplementary Method 1 2

Supplementary Figures

Supplementary Fig. 1 4

Supplementary Fig. 2 5

Supplementary Fig. 3 6

Supplementary Fig. 4 7

Supplementary Fig. 5 8

Supplementary Fig. 6 9

Supplementary Tables

Supplementary Table 1 10

Supplementary Table 2 11

Supplementary Table 3 12

Supplementary Table 4 13

Supplementary Table 5 14

Supplementary Table 6 15

Supplementary Table 7 16

Supplementary References 17

Supplementary Method 1

Methods for path-based comparison models. Random walk generated path-based models leverage skip-gram model for node representation learning. All models including proposed model DREAMwalk generated node embedding vectors through same Skip-gram[1] settings from sampled paths. For all models, 100 paths of length 10 were samples for each node. Latent vector dimension was set to size of 128.

node2vec. node2vec[2] is a flexible random walk generated path-based method that balances between breadth-first sampling and depth-first sampling through parameters p and q. For all experiments, p and q were set to 1. A logistic regression classifier was applied for drug-disease association (DDA) prediction.

edge2vec. edge2vec[3] is a node2vec-based model that considers heterogeneous edge types and performs biased path generation. Prior to biased path sampling, edge2vec first performs uniform path sampling for learning the edge type distribution of the network and generate an edge-type transition matrix. For edge-type transition matrix generation process, 1 path of length 10 were sampled for all nodes. Also, bias parameters p and q were set to 1. A logistic regression classifier was applied for drug-disease association (DDA) prediction, following the authors' proposal.

Methods for similarity-based comparison models. Several drug repurposing models leverage different similarity networks for drug-disease association (DDA) prediction. Two state-of-the-art biomedical knowledge graph link prediction methods, DTi2vec[4] and NEWMIN[5], were selected for DDA performance comparison. The similarity networks used as input for the two models are semantic similarity network and biological target similarity network.

Biological target similarity network. For biological target similarity network, Jaccard similarity measure was utilized for generating drug-drug and disease-disease similarity networks based on their associated gene sets. Drug-drug network was generated from drug-target protein bipartite network, and disease-disease network was generated from disease-gene association bipartite network in each biomedical knowledge graphs.

Semantic similarity network. The measure of Jiang and Conrath[6], modified by Seco et al.[7], was utilized for generating semantic similarity network of drug and disease from ontologies or hierarchy of disease and drugs.

DTi2vec[4]. DTi2vec is a biomedical knowledge graph embedding and link prediction method that was originally developed for predicting drug-target interactions on biomedical knowledge graphs. While the original framework generates drug-drug and protein-protein similarity networks for drug-protein interaction prediction, the model can be extended to predict DDA by utilizing the drug-drug and disease-disease similarity networks. The method constructs a similarity-based bipartite knowledge graph, which is refined through a k-nearest-neighbor (KNN) - based framework. Following this procedure, a single weighted similarity network is constructed. To generate node embeddings, the method uses node2vec-based random walk and skip-gram algorithms. Finally, DDA prediction is performed using an eXtreme Gradient Boosting (XGBoost)[8] classifier, which utilizes the concatenated vector of drug and disease embeddings as input feature.

NEWMIN[5]. NEWMIN is another biomedical knowledge graph embedding and link prediction methods that was originally proposed for drug combination prediction. Instead of combining multiple similarity graphs into one representative weighted knowledge graph, NEWMIN performs random walks on all of the knowledge graphs. While doing so, the model adapts and performs random walks with different numbers on each network determined by which network is the most critical for downstream tasks. In other words, random walk-based sequence generation is performed the most on the network that contributes the most to performance increase. After generating the node sequences, Skip-gram algorithm is adopted for node embedding generation. Then, a Random Forest classifier receives the concatenated embedding vectors for association prediction.

Methods for GNN-based comparison models. Unlike path-based models, link prediction using graph neural network (GNN) models are performed in an end-to-end manner. Two state-of-the-art GNN-based link prediction models, namely SEAL[9] and WalkPool[10], were used for performance comparison.

SEAL[9]. SEAL is a subgraph-based link prediction model that utilizes the concept of heuristics and applies them to be learned through a GNN. Especially, the model approximates the heuristics of the whole network from an *h*-hop local enclosing subgraph. The model is designed to be able to include a node2vec embeddings in the node features. For our experiments, we included the 128-dimension node2vec embeddings as node features and adopted default parameters of the proposed version. Also, for the model to be aware of the different node types, the one-hot vector of the node type was concatenated to the node features. The model was trained with 10-epoch early stopping criteria using a validation set.

WalkPool[10]. WalkPool is a state-of-the-art link prediction model that is also based on a h-hop local enclosing subgraph method. WalkPool extracts higher-order graph topology information through random-walk generated walk profiles on latent graph. WalkPool is also designed to be able to include a pre-trained node feature. For our experiments, we included the 128-dimension node2vec embeddings, along with the one-hot vector of the node type concatenated to the node features. We adopted the default parameters from the proposed model. The model was trained with 10-epoch early stopping criteria using a validation set.

Methods for transition-based comparison models. Another category of state-of-the-art models for knowedge graph link prediction is transition-based models. These models regard the associations in a knowledge graph as transitions from the source entity to the target entity. ComplEx[10], RotatE[11] and QuatE[12] are transition-based models with different modeling of the embedding space. ComplEx demonstrated that learning low-dimensional representation of entities and relations on the complex space is highly effective with the asymmetrical Hermitian product as the relation operation. RotatE models the relation of entities as rotation on a single plane of complex space, and QuatE extended the rotation on the hypercomplex spaces with two planes of rotation.

Since the transition-based approaches learn the representations of entities and relations altogether from the given knowledge graph, we included all types of relations in the knowledge graph in the train set and left the drug-disease associations for validation and test sets. All models were trained with default parameters, with 10-epoch early stopping criteria using a valid set.

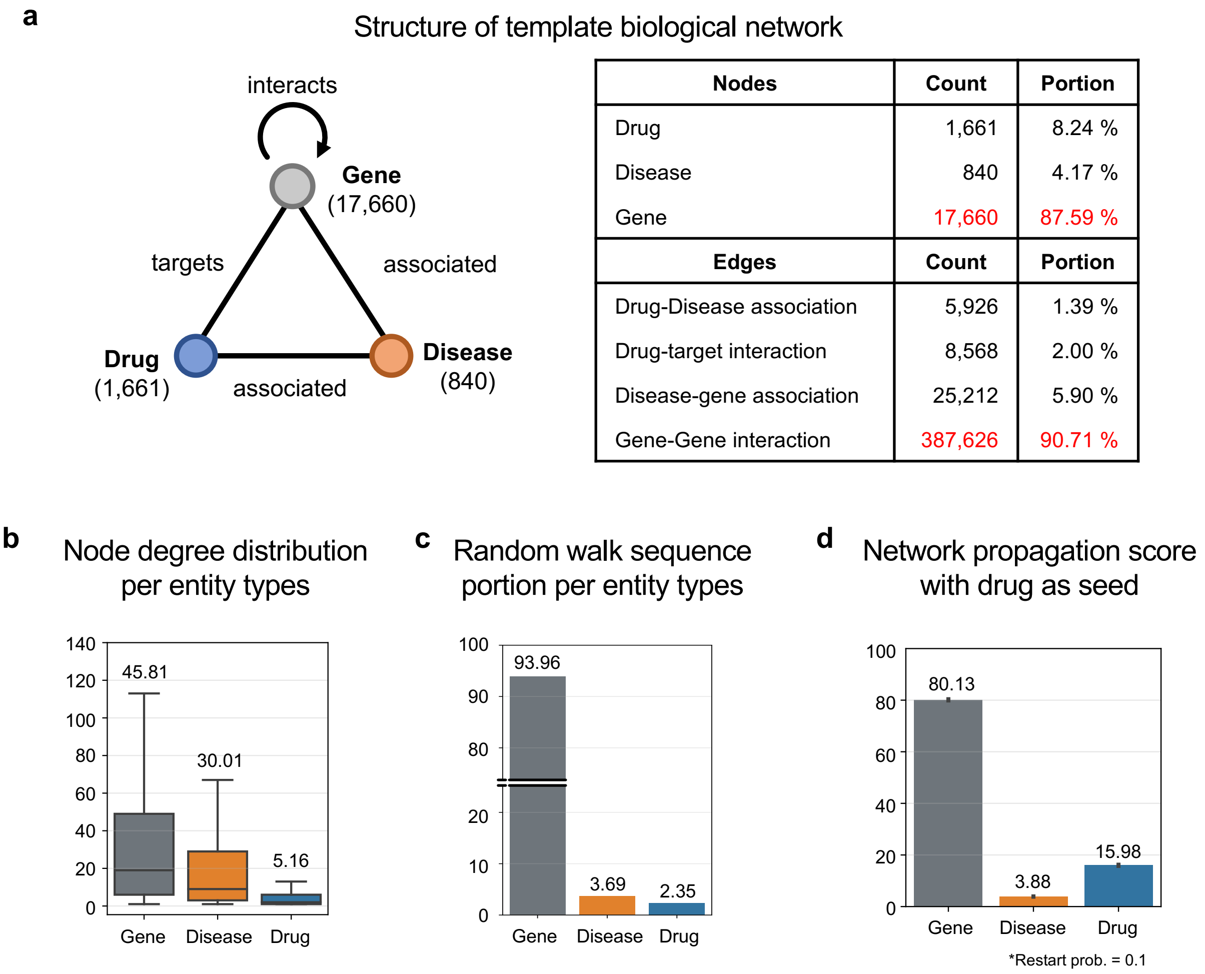

Nodes	Count	Portion
Drug	1,661	8.24 %
Disease	840	4.17 %
Gene	17,660	87.59 %
**Edges**	**Count**	**Portion**
Drug-Disease association	5,926	1.39 %
Drug-target interaction	8,568	2.00 %
Disease-gene association	25,212	5.90 %
Gene-Gene interaction	387,626	90.71 %

Supplementary Fig. 1 Analysis of template drug-gene-disease network reveals its bias to gene-gene network. a Schematic and statistics of template drug-gene-disease network, extracted from the MSI[11] network. Genes cover up over 87% of the nodes and gene-gene interaction edges cover up over 90% of the edges on the whole network. **b** Node degree distribution for each node types. The degrees of gene nodes are higher (average 45.8) than that of drugs (5.2) and diseases (30.0). On the box plot, the center line represents the median, while the upper and lower box limits denote the upper and lower quartiles, respectively. The whiskers indicate 1.5 times the interquartile range. The box plots have been derived from independent n=17,660 gene, n=1,661 drug and n=840 disease entities. **c** Node type distribution from sampled random walk sequences. 10 walks of length 10 were generated for each node uniformly. **d.** Network propagation scores for each node type, performed with Random walk with Restart (RWR) algorithm. The RWR was performed for all drug entities as seeds with restart probability of 0.1. The average score is notated above each plot. The error bars denote the mean values +/- 95% confidence interval, derived through n=1,661 independent experiments. Source data are provided as a Source Data file. (Restart prob.: restart probability for RWR algorithm.)

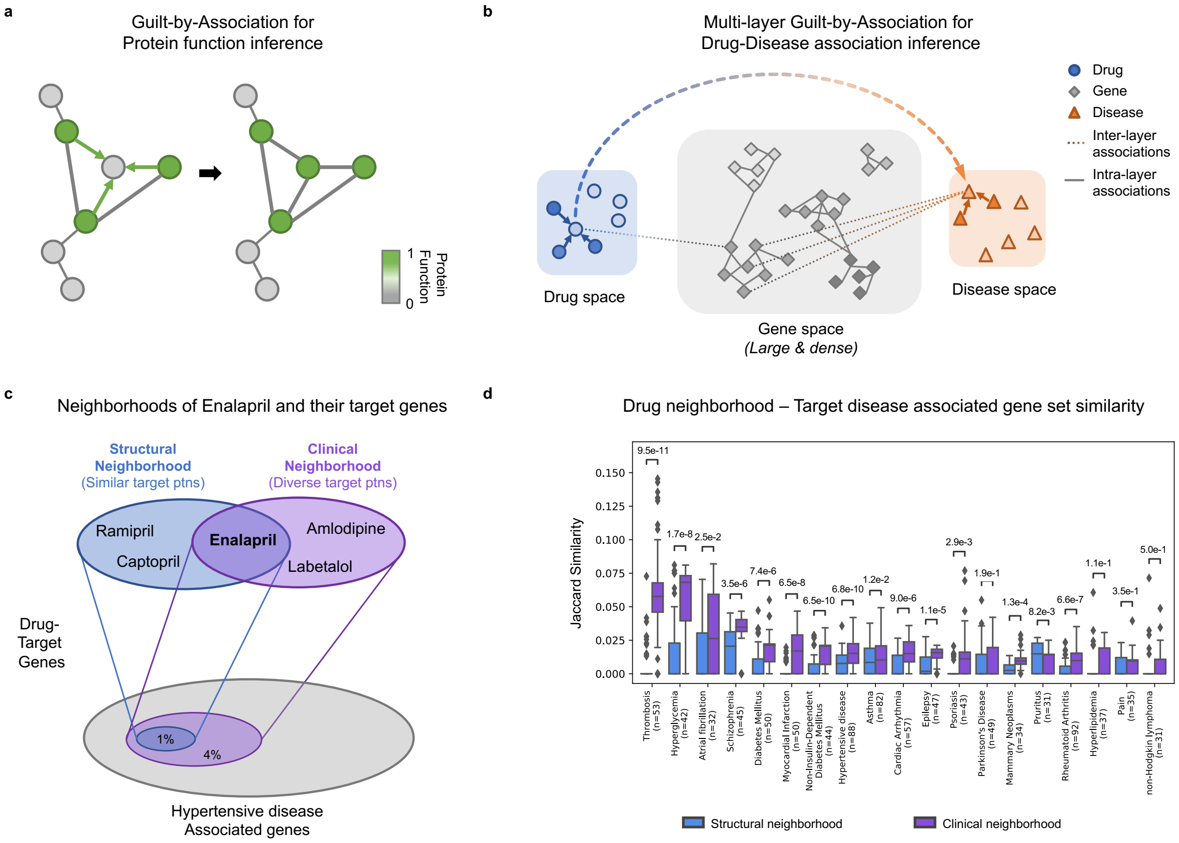

Supplementary Fig. 2. Multi-layer Guilt-by-Association (GBA) and network analysis of drug neighborhoods revealing protein targets of a semantic drug neighborhood cover a wider area of target disease-associated genes. a GBA for protein function inference through looking at a protein's interaction neighbors on a single layer. **b** Multi-layer GBA for drug-disease association inference through looking at a drug's neighbors. **c** Concept of hypertensive drug enalapril's structural neighborhood and semantic neighborhood, and their target genes relative to hypertensive disease-associated genes. **d** Gene set similarity of neighborhood target genes and disease-associated genes. Target genes of semantic neighborhood show higher similarity with target disease-associated genes compared to structural neighborhood. On the box plots, the center line represents the median, while the upper and lower box limits denote the upper and lower quartiles, respectively. The whiskers indicate 1.5 times the interquartile range. The numbers above each box plot pair summarizes the resulting *p*-values of two-sided paired t-tests. No multiple test adjustments have been performed during the *p*-value calculation. The number of independent drug-disease pair data are provided next to corresponding disease names (n=X). Source data are provided as a Source Data file.

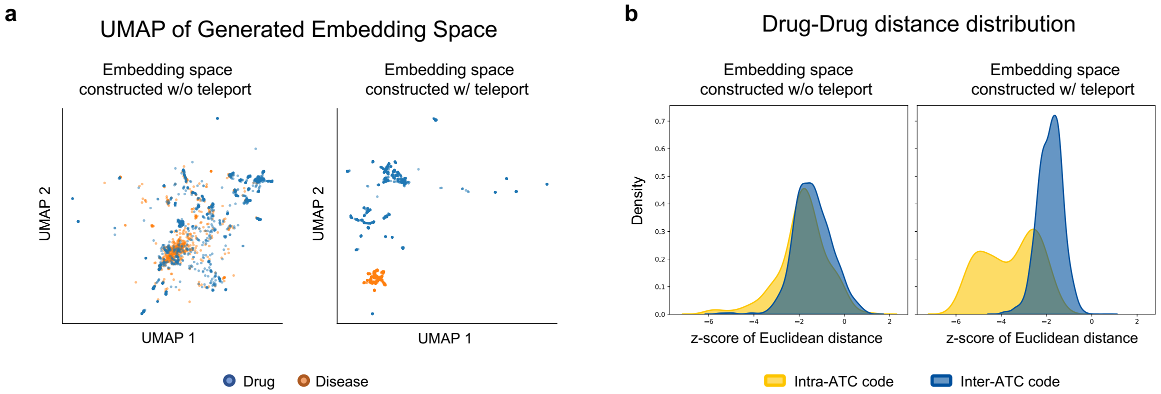

Supplementary Fig. 3. Embedding space of DREAMwalk exhibits its ability to distinguish entities based on their semantics. a The UMAP plot of without-teleport embedding space (left) and DREAMwalk embedding space generated with teleport (right). Drug (blue) and disease (orange) entities are well separated in the DREAMwalk's embedding space. **b** Comparison of intra-ATC level 1 distances (yellow) and inter-ATC level 1 distances (blue) on DREAMwalk embedding space (left) and without-teleport embedding space (right). Intra-ATC code distance refers to the distances between the source and target drugs in the same first-level ATC code, whereas inter-ATC code distance refers to the distance between drugs of different first-level ATC codes. Since the first-level ATC code clusters drugs into 14 main anatomical or pharmacological groups, the multi-layer GBA space shows drugs sharing ATC annotations located closer to each other. In contrast, the distance distribution of the space generated without teleport implicit the biological level network contains insufficient information for clustering drugs based on their anatomical and pharmacological characteristics. Source data are provided as a Source Data file. (ATC code: Anatomical Therapeutic code; UMAP: Uniform Manifold Approximation and Projection)

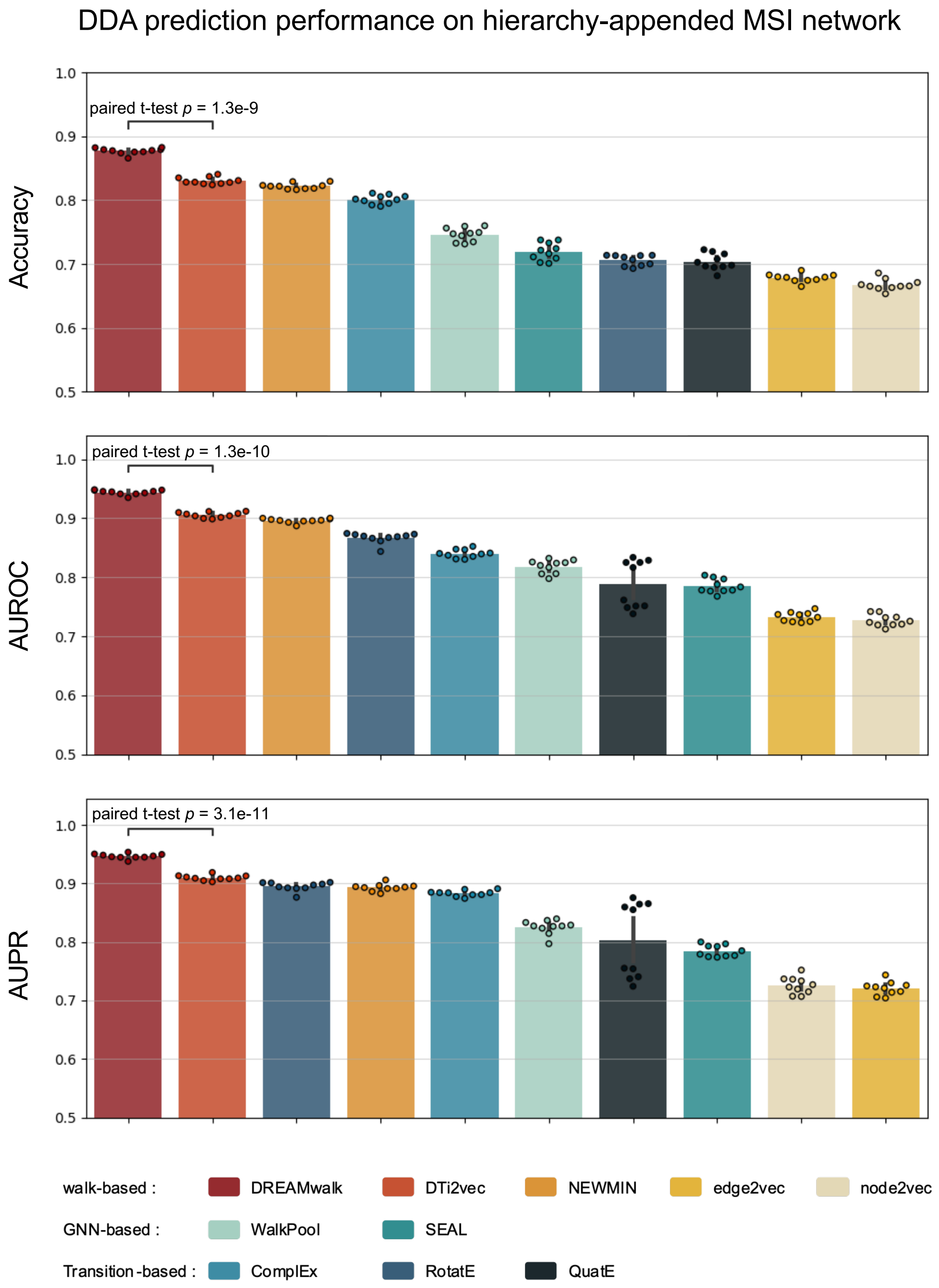

Supplementary Fig. 4. Drug-disease association prediction performances of comparison models on hierarchy-appended MSI network. The error bars denote the mean values +/- 95% confidence interval, derived through n=10 independent experiments. The notation above each box plot represents the resulting *p*-values of the two-sided paired t-test between DREAMwalk and the next best-performing model, DTi2vec. Source data are provided as a Source Data file. (AUROC: Area Under the Receiver-Operating Characteristics curve; AUPR: Area Under the Precision-Recall curve; DDA: drug-disease association, paired t-test *p*: resulting *p*-value of the two-sided paired t-test)

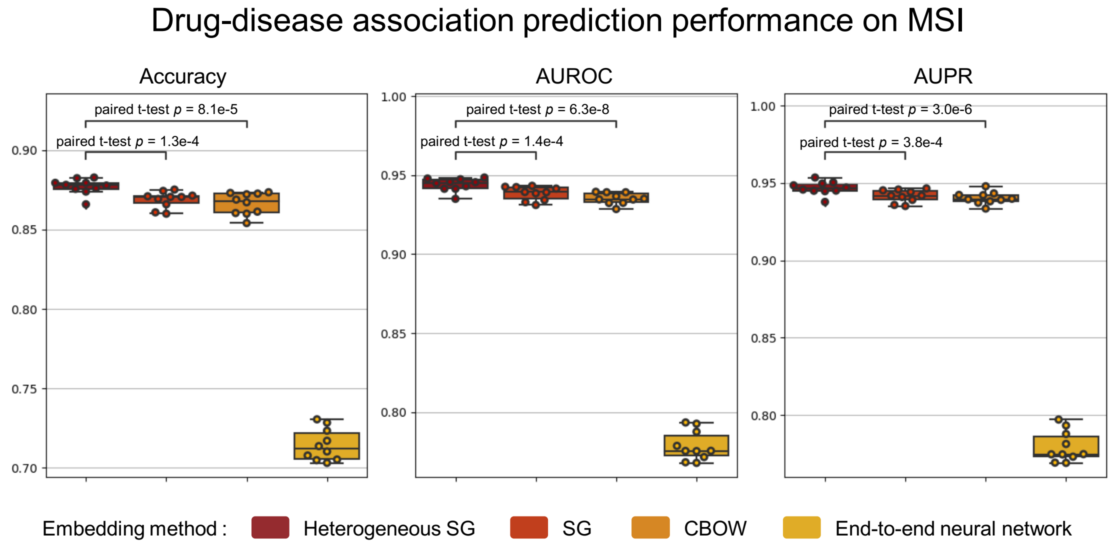

Supplementary Fig. 5. Drug-disease association prediction performances following the change in embedding method on the MSI network. Prediction performance of Heterogeneous SG is significantly higher than that of SG[1], CBOW[1] and end-to-end neural network embedding methods. On the box plots, the center line represents the median, while the upper and lower box limits denote the upper and lower quartiles, respectively. The whiskers indicate 1.5 times the interquartile range. All data have been derived through n=10 independent experiments. The notation above each box plot represents the resulting *p*-values of two-sided paired t-tests. Source data are provided as a Source Data file. (AUROC: Area Under the Receiver-Operating Characteristics curve; AUPR: Area Under the Precision-Recall curve; CBOW: Continuous Bag-of-words; SG: Skip-gram; paired t-test *p*: the resulting *p*-value of two-sided paired t-test)

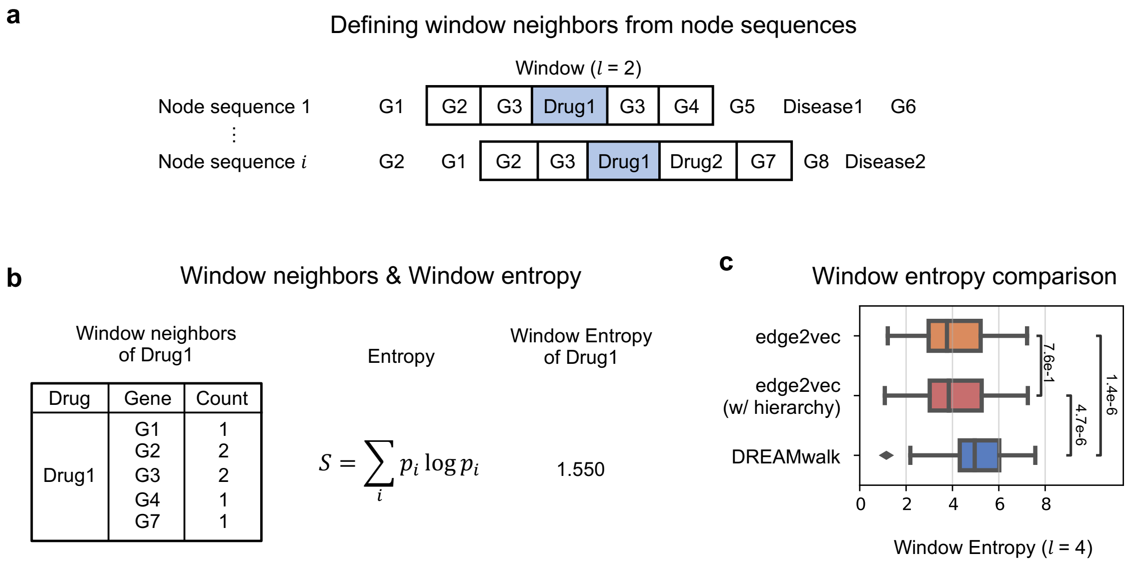

Supplementary Fig. 6. Window neighbor entropy comparison of edge2vec and DREAMwalk. **a** Selection of from node sequences with window length of 2. **b** Calculating window entropy from generated node sequences based on each neighbor's counts. **c** Comparison of window entropy for all drug entities with node sequences generated by edge2vec (on original MSI network), edge2vec w/ hierarchy (on MSI network with hierarchy entities as nodes), and DREAMwalk. The window length was set to 4 for window entropy comparison. On the box plots, the center line represents the median, while the upper and lower box limits denote the upper and lower quartiles, respectively. The whiskers indicate 1.5 times the interquartile range. All data have been derived through n=100 independent entities for each model. The numbers above each box plot pair summarizes the resulting *p*-values of two-sided t-tests. No multiple test adjustments have been performed during the *p*-value calculation. Source data are provided as a Source Data file.

Network	Hierarchy	Number of terms	Number of associations	Total levels
**MSI**	ATC	4,647	4,543	5
	MeSH	3,328	4,373	12
**Hetionet**	ATC	3,289	3,274	5
	DO	308	302	12
**KEGG**	ATC	5,314	5,210	5
	ICD-11	1,126	972	7

Supplementary Table 1. Statistics of utilized drug/disease hierarchies. ATC classification, MeSH term, DO and ICD-11 hierarchies for each of the three heterogeneous networks.

Nodes	
Drugs	1,661
Diseases (Indications)	840
Genes	17,660
GO Molecular Functions	9,798
**Edges**	
Drug-disease associations	5,926
Drug-target interactions	8,568
Disease-gene associations	25,212
Protein-protein interactions	387,626
Gene-GO Molecular Function annotations	34,777
GO Molecular Function associations	22,545

Supplementary Table 2. Statistics of the MSI network. All the nodes and edges of the original MSI[14] network are utilized for experiments.

Nodes	
Compounds (drugs)	1,552
Diseases	137
Genes	20,945
Pathways	1,822
**Edges**	
Compound-treats-Disease	755
Compound-binds-Gene	11,571
Compound-upregulates-Gene	18,756
Compound-downregulates-Gene	21,102
Disease-associates-Gene	12,623
Disease-upregulates-Gene	7,731
Disease-downregulates-Gene	7,623
Gene-regulates-Gene	265,672
Gene-interacts-Gene	147,164
Gene-covaries-Gene	61,690
Gene-participates-Pathway	84,372

Supplementary Table 3. Statistics of the HetioNet network. Nodes and edges associated with drugs, diseases, genes were extracted from the original Hetionet[15] to construct a biological level network.

Nodes	
Compounds (drugs)	6,008
Diseases	1,963
Genes	14,496
Pathways	461
**Edges**	
Drug-disease association	2,272
Disease-gene association	6,319
Drug-gene association	11,860
Gene-pathway association	43,226
Pathway-pathway association	2,129
Disease-pathway association	2,573
Drug-pathway association	10,274

Supplementary Table 4. Statistics of the KEGG network. Nodes and edges associated with drugs, diseases, genes were extracted from the KEGG[16] network.

Alzheimer’s disease						
**Rank**	**DTi2vec**	**NEWMIN**	**WalkPool**	**SEAL**	**ComplEx**	**edge2vec**
1	Promazine	Clonazepam	Isoflurophate	Zinc	Dopamine	Dexamethasone
2	Melatonin	Carbamazepine	Mestinon	Cyclophosphamide	Amantadine	Hydrocortisone
3	Felbamate	Propentofylline	Choline	Ritonavir	Phenobarbital	Prednisolone
4	Tetrabenazine	Sertraline	Choline salicylate	Rasagiline	Norepinephrine	Triamcinolone
5	Gabapentin	Topiramate	Dipivefrine	Melatonin	Ifosfamide	Interferon alfa-2b
6	Diazepam	Tiagabine	Mecasermin	Resveratrol	Leuprolide	Fludroxycortide
7	Lorazepam	Levetiracetam	Edrophonium	Mecasermin	Theophylline	Prednisone
8	Phenytoin	Ramelteon	Pralidoxime	Tranylcypromine	Cisplatin	Rimexolone
9	Clonazepam	Retigabine	Pralidoxime-chloride	Diacerein	Baclofen	Methylprednisolone
10	Methamphetamine	Chlordiazepoxide	Demecarium	Choline salicylate	Orphenadrine	Buphenine

Breast Carcinoma						
**Rank**	**DTi2vec**	**NEWMIN**	**WalkPool**	**SEAL**	**ComplEx**	**edge2vec**
1	Canakinumab	Interferon alfa-2b	Thalidomide	Succinic acid	Etoposide	Dexamethasone
2	Methotrexate	Vinblastine	Fostamatinib	Aspirin	Vincristine	Hydrocortisone
3	Interferon alfa-2b	Peginterferon alfa-2a	Dexrazoxane	Arsenic trioxide	Hydroxyurea	Triamcinolone
4	Vinblastine	Carmustine	Teniposide	Dexamethasone	Irinotecan	Prednisolone
5	Thalidomide	Interferon Alfa-2a	Etoposide	Arsenic-trioxide	Cisplatin	Prednisone
6	Thiotepa	Vincristine	Daunorubicin	Prednisolone	Dactinomycin	Interferon alfa-2b
7	Methylprednisolone	Interferon alfacon-1	Clofarabine	Hydrocortisone	Vinblastine	Fludroxycortide
8	Cisplatin	Hydroxyurea	Dexamethasone	Amcinonide	Bleomycin	Buphenine
9	Interferon beta-1a	Interferon beta-1a	Dactinomycin	Urea	Carmustine	Methylprednisolone
10	Azathioprine	Peginterferon alfa-2b	Cytarabine	Prednisone	Ifosfamide	Interferon beta-1b

Supplementary Table 5. Top-10 drug repurposing candidates for Alzheimer’s disease and breast carcinoma of baseline models.

Breast Carcinoma				
**Rank**	**Drug**	**Original indication**	**Avg. Prob.**	**SD**
1641	Lacosamide	Epilepsy, seizures	0.0012	0.00079
1642	Phenacemide	Epilepsy, Epilepsy, Complex Partial, seizures	0.0012	0.00087
1643	Ajmaline	Cardiac Arrhythmia	0.0011	0.00118
1644	Enoximone	congestive heart failure	0.0011	0.00086
1645	Molsidomine	coronary artery disease	0.0011	0.00090
1646	Encainide	Premature Ventricular Contractions, Cardiac Arrhythmia	0.0010	0.00097
1647	Tocainide	ventricular arrhythmias, Cardiac Arrhythmia	0.0010	0.00078
1648	Mephenytoin	seizures	0.0009	0.00058
1649	Moricizine	ventricular arrhythmias, Cardiac Arrhythmia	0.0008	0.00095
1650	Vernakalant	Cardiac Arrhythmia, atrial fibrillation	0.0006	0.00066
**Alzheimer's disease**				
**Rank**	**Drug**	**Original indication**	**Avg. Prob.**	**SD**
1641	Enoxacin	Urinary tract infection, Cystitis	0.0062	0.00525
1642	Itraconazole	onychomycosis	0.0061	0.00582
1643	Benralizumab	asthma	0.006	0.00747
1644	Penicillin-v-potassium	Erysipelas, Gingivostomatitis, pneumonia	0.0059	0.00389
1645	Ofatumumab	chronic lymphocytic leukemia	0.0058	0.00737
1646	Lomefloxacin	Urinary tract infection, Lower respiratory tract infection, tuberculosis	0.0053	0.00416
1647	Sparfloxacin	Pneumonia, tuberculosis	0.0044	0.00474
1648	Pyrazinamide	tuberculosis	0.0039	0.00314
1649	Trovafloxacin	Urinary tract infection, sinusitis	0.0039	0.00340
1650	Fluconazole	meningitis	0.0032	0.00290

Supplementary Table 6. Bottom-10 list of the drug repurposing candidates by DREAMwalk for Breast carcinoma and Alzheimer's disease. (Avg. Prob: average probability; SD : Standard deviation)

Repurposing case	DREAMwalk	NEWMIN	DTi2vec	SEAL	WalkPool	node2vec	edge2vec	ComplEx
**Aspirin-MI**	**0.9987**	0.9946	0.9949	0.9171	0.9504	0.7559	0.567	0.692
**Aspirin-Thrombosis**	**0.9864**	0.9844	0.9695	0.8746	0.8276	0.7229	0.6923	0.4608
**Sildenafil-ED**	**0.9523**	0.6174	0.4708	0.6958	0.632	0.4309	0.6299	0.3345
**Sildenafil-PAH**	0.3554	0.5247	0.4764	0.4202	0.4382	0.4170	**0.6695**	0.1665
**Thalidomide-MM**	0.9772	**0.9964**	0.9896	0.9129	0.6886	0.5986	0.4951	0.6499
**Thalidomide-RCC**	0.6023	0.8847	**0.9632**	0.8237	0.6539	0.6569	0.4659	0.4896
**Finasteride-Male Alopecia**	**0.4087**	0.2413	0.2824	0.0927	0.2326	0.3179	0.3072	0.172
**Minoxidil-Alopecia**	0.1478	0.1451	0.2398	0.1203	0.161	**0.3740**	0.3205	0.3427
**Median**	**0.7773**	0.7511	0.7198	0.7598	0.6430	0.5148	0.5311	0.4018

Supplementary Table 7. Predicted probabilities of each model on actual drug repurposing cases. The drug repurposing cases are based on the review by Jourdan *et al.* (2020). The highest probability for each drug-disease pair is highlighted in bold. (ED: Erectile dysfunction; MI: Myocardial infarction; MM: Multiple Myeloma; PAH: Pulmonary arterial hypertension; RCC: Renal Cell Carcinoma)

Supplementary References

1. Mikolov, Tomas, et al. "Efficient estimation of word representations in vector space." arXiv preprint arXiv:1301.3781 (2013).
2. Grover, Aditya, and Jure Leskovec. "node2vec: Scalable feature learning for networks." *Proceedings of the 22nd ACM SIGKDD international conference on Knowledge discovery and data mining*. 2016.
3. Gao, Zheng, et al. "edge2vec: Representation learning using edge semantics for biomedical knowledge discovery." *BMC bioinformatics* 20.1 (2019): 1-15.
4. Thafar, Maha A., et al. "DTi2Vec: Drug–target interaction prediction using network embedding and ensemble learning." *Journal of cheminformatics* 13.1 (2021): 1-18.
5. Yu, Liang, Mingfei Xia, and Qi An. "A network embedding framework based on integrating multiplex network for drug combination prediction." *Briefings in bioinformatics* 23.1 (2022): bbab364.
6. Jiang, J. J. & Conrath, D. Semantic similarity based on corpus statistics and lexical taxonomy. In *Proceedings of the 10th International Conference on Research in Computational Linguistics*, *ROCLING*, vol. 97 (1997).
7. Seco, Nuno, Tony Veale, and Jer Hayes. "An intrinsic information content metric for semantic similarity in WordNet." *Ecai*. Vol. 16. 2004.
8. Chen, Tianqi, and Carlos Guestrin. "Xgboost: A scalable tree boosting system." *Proceedings of the 22nd acm sigkdd international conference on knowledge discovery and data mining*. 2016.
9. Zhang, Muhan, and Yixin Chen. "Link prediction based on graph neural networks." *Advances in neural information processing systems* 31 (2018).
10. Pan, Liming, Cheng Shi, and Ivan Dokmanić. "Neural link prediction with walk pooling." In *International Conference on Learning Representations (2022).*
11. Trouillon, Théo, et al. "Complex embeddings for simple link prediction." *International conference on machine learning*. PMLR, 2016.
12. Sun, Zhiqing, et al. "Rotate: Knowledge graph embedding by relational rotation in complex space." In *International Conference on Learning Representations* (2019).
13. Zhang, Shuai, et al. "Quaternion knowledge graph embeddings." *Advances in neural information processing systems* 32 (2019).
14. Ruiz, Camilo, Marinka Zitnik, and Jure Leskovec. "Identification of disease treatment mechanisms through the multiscale interactome." *Nature communications* 12.1 (2021): 1-15.
15. Himmelstein, Daniel Scott, et al. "Systematic integration of biomedical knowledge prioritizes drugs for repurposing." *Elife* 6 (2017): e26726.
16. Kanehisa, Minoru, et al. "KEGG for linking genomes to life and the environment." *Nucleic acids research* 36.suppl_1 (2007): D480-D484.
17. Jourdan, Jean-Pierre, et al. "Drug repositioning: a brief overview." *Journal of Pharmacy and Pharmacology* 72.9 (2020): 1145-1151.
